# Modernization Trends of Infertility Treatment of Traditional Korean Medicine

**DOI:** 10.1155/2017/4835912

**Published:** 2017-11-05

**Authors:** Jang-Kyung Park, Dong-Il Kim

**Affiliations:** ^1^College of Korean Medicine, Dongguk University, Seoul, Republic of Korea; ^2^Department of Obstetrics and Gynecology, College of Korean Medicine, Dongguk University, Seoul, Republic of Korea

## Abstract

Despite the development of assisted reproductive technology (ART), it is difficult to increase the implantation rate. In Korea, Traditional Korean Medicine, including herbal medicine, is an important component of infertility treatment. Korean medical doctors who are treating infertility often use herbal medicine to promote implantation. In this article, as one of the research works on modernization of Traditional Korean Medicine, we investigated the experimental studies to clarify the effects of herbal medicines that are traditionally used to promote pregnancy. We searched for experimental studies over the past 10 years of improvement of endometrial receptivity in herbal medicine using six domestic and international sites. We analyzed 11 studies that meet the selection criteria. We found that herbal medicines demonstrably improved endometrial receptivity and increased pregnancy rates.

## 1. Introduction

The modernization of Traditional Korean Medicine means combining or converting it into modern technology and modern scientific culture, which includes studies of the efficacy, effect, and mechanism of herbal medicine [1–3].

In 2012, South Korea's infertility rate is 32.3%, an annual average growth rate of 7.7%. The overall fertility rate is the lowest in the world, causing a serious social problem [4]. Despite advances in assisted reproductive technology (ART) and national support program for infertile couples, the success rate remains 25% to 30% because of implantation dysfunction [5]. Infertile couples in South Korea often turn to Traditional Korean Medicine as an important component of primary or secondary treatment of infertility [6].

The implantation process is through the interaction of the embryo and the endometrium and occurs in a very limited period of time, called the window of implantation. Ovarian stimulation, though essential for IVF, may itself have detrimental effects on endometrial receptivity, embryonic implantation, and perhaps pregnancy outcomes [7].

As part of the study on modernization of Korean Medicine, we reviewed experimental studies of endometrial receptivity in herbal medicine. Studies on embryo implantation are mainly focused on experimental studies because they can cause ethical problems; therefore, only experimental studies were selected in this study [8].

Through review of studies, we tried to clarify the effect of the herbal medicine on the endometrial receptivity and ascertain the significance as an effective treatment modality for infertility.

## 2. Materials and Methods

### 2.1. Data Sources and Searches

We searched experimental studies in six domestic and foreign search sites (NDSL, KSTUDY, RISS, OASIS, JKOM, and MEDLINE). With June 1, 2017, as the end date, the study was limited to the last 10 years, and the language was limited to Korean and English. In domestic search sites, we searched for the keywords “endometrial receptivity”, “Traditional Korean Medicine”, and “Herbal medicine”. In Medline, we used the mesh term of “medicine, east Asian traditional”, “medicine, Chinese traditional”, “medicine, Korean traditional”, “medicine, Kampo”, “plant extracts”, “herbal medicine”, “uterine receptivity”, “uterine endometrium”, “endometrial receptivity”, and “embryo implantation”. We searched for each of the terms and finally searched for intersection combination.

### 2.2. Study Selection

**Figure 1:**
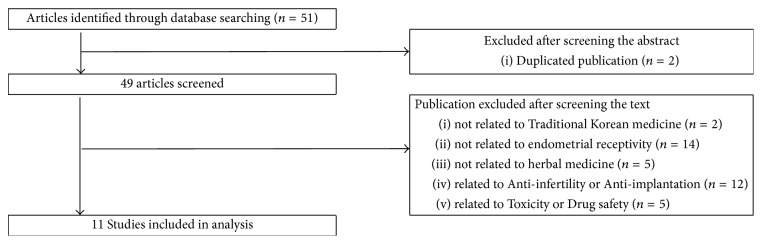
The process of data selection and extraction.

We searched experimental studies of the positive effect of endometrial receptivity in herbal medicine. Exclusions included studies of the mechanism by which Traditional Korean Medicine interferes with pregnancy and safety and toxicity studies. Studies where the intervention was not herbal medicine and where the subject was not endometrial receptivity were also excluded (Figure 1).

## 3. Results

### 3.1. Study Description

No articles were found in domestic search sites. In Medline, 51 studies were found. Out of these, 11 studies were selected. Two of the authors (PJK, KDI) read the title and abstract and reviewed the 51 initial papers. After selecting 49 papers, excluding two duplicate papers, two authors (PJK, KDI) reviewed the original articles and extracted the key information. When opinions differed, the decision was agreed through discussion.

### 3.2. Analysis of the Publishing Year

The review found that one paper on the topics of interest was published yearly from 2008 to 2014, and two were published yearly in 2015 and 2016.

### 3.3. Analysis of the Publishing Country

Ten out of eleven studies were from China, and one was from South Korea.

### 3.4. Analysis of Study Design

#### 3.4.1. Control Group

In all 11 studies, in vivo studies were performed rather than in vitro. All 11 studies included in vivo studies; Choi et al. [9] conducted both in vivo and in vitro studies.

Each study included at least two control groups, including a normal group and model group. The models were either COH induction or EID induction. Embryo implantation dysfunction (EID) model was used in six studies [6, 9–13] and controlled ovarian hyperstimulation (COH) model in four studies [14–17]. In one study, both EID model and COH model were examined [18]. In two studies, a positive control (progynova [10], aspirin [16]) was also established. To induce the COH model, pregnant mare serum gonadotropin (PMSC) and human chorionic gonadotropin (hCG) were used. Mifepristone was used in four cases and indomethacin in three studies to derive EID. Li et al. described a COH model, but it was judged to be an EID model induced by indomethacin [12].

#### 3.4.2. Outcome Measures

Immunohistochemical assay, real time polymerase chain reaction (RT-PCR) assay, TUNEL method, biomarkers associated with endometrial receptivity such as endometrial thickness, angiogenesis, and pinopodes by microscopic examination were measured by microsocpic exam. Polypeptides associated with endometrial receptivity and microscopic observation with pregnancy were the most frequently used indicators of evaluation. Number of implanted blastocysts, pregnancy rate, and expression of integrin *β*3 mRNA were used in four studies. Expression of LIF mRNA was used in three studies, expression of LIF protein, and implantation sites were used in two studies, and COX-2, IFN-*γ*, IL-10, integrin *αν* mRNA, integrin *β*3 proteins, LIF mRNA/*β*-actin, MMP-9, NF-*κ*ВOPN mRNA, PGI2, PPARd, IL-11 mRNA, TIMP-3, apoptotic index, proliferative index, LCM-DE-MS, microvessel density, endometrial thickness, and pinopodes in the epithelium were used in one study (Table 1).

### 3.5. Analysis

In all 11 studies, herbal medicines improved endometrial receptivity compared to the model group. There was an improvement in the expression of polypeptides related to endometrial receptivity compared to aspirin and progynova (Table 1).

### 3.6. Herbal Medicine

In one study, the efficacy of a single herbal medicinal material was assessed, and, in ten studies, the efficacy of a prescription consisting of a herbal combination was evaluated.

#### 3.6.1. Herbal Formula

Bushenantai prescription was used in two studies [6, 14], and Xianziyizhen [10], Bushenyiqihexue [11], DS-1-47 [12], Bangdeyun [13], Er'zhi Tiangui [15], Shoutaiwai [16], Yiqixue Buganshen [17], and Zhuyun [18] prescriptions were each used in one study (Table 2).

Eight out of ten studies using combination prescriptions described the Oriental Medicinal efficacy of the prescription. Among the efficacy of prescription, invigorating or tonifying the kidney [6, 10–14, 16, 17] was the most common, followed by replenishing or supplement Qi [11–13, 17], nourishing blood [6, 13, 17], and activating or promoting blood [6, 11, 14].

#### 3.6.2. Herbal Medicinal Materials

**Figure 2:**
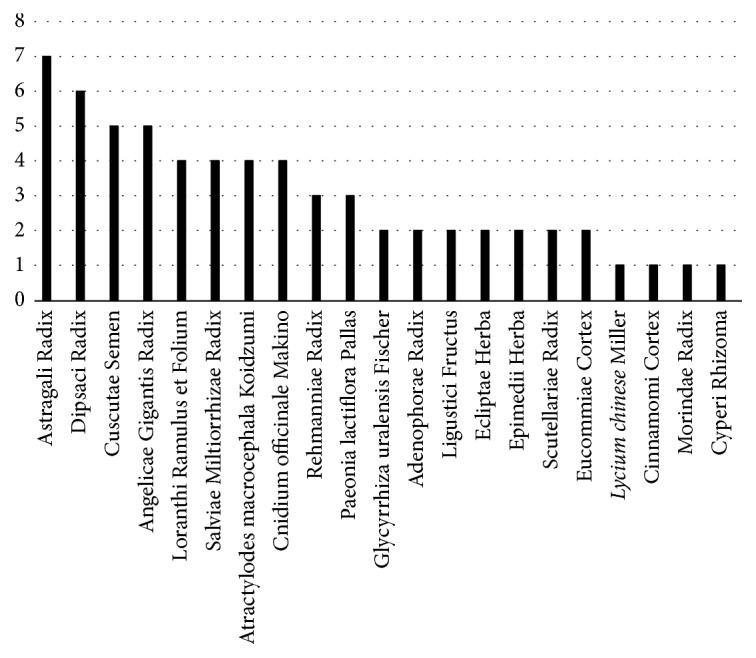
*Kinds and Frequencies of Medicinal Materials.* Among the 11 studies, the most used herb was Astragali Radix which was included in 7 studies, followed by Dipsaci Radix included in 6 studies, Cuscutae Semen and Angelicae Gigantis Radix included in 5 studies, Loranthi Ramulus et Folium, Salviae Miltiorrhizae Radix,* Atractylodes macrocephala* Koidzumi, and Cnicium officinale Makino included in 4 studies, and Rehmanniae Radix and Paeonia lactiflora Pallas included in 3 studies.

Twenty-two herbal medicinal materials were used in 11 studies. The most frequently used herbal medicinal material was Astragali Radix, followed by Dipsaci Radix, Cuscutae Semen, and Angelicae Gigantis Radix (Figure 2).

**Figure 3:**
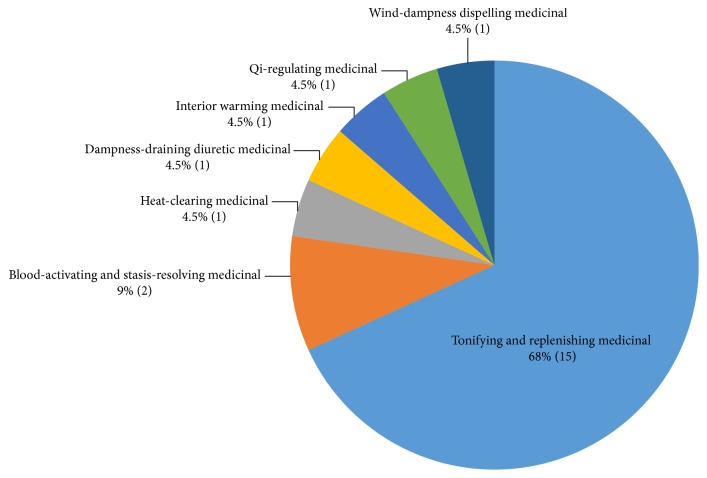
*Classified by Single Medicinal Materials Type.* According to oriental herbal efficacy classification criteria, the herb medicinal belonging to tonifying and replenishing medicinal was the most common with 15 species. It is followed by blood activating and stasis-resolving medicinal, heat-clearing medicinal, dampness-draining diuretic medicinal, interior-warning medicinal, qi-regulating medicinal, and wind-dampness dispelling medicinal.

By oriental herbal efficacy [19], the highest rate was for the tonifying and replenishing medicinal effects (68%). Among the tonifying and replenishing medicinals, five yang-tonifying medicinals, four yin-tonifying medicinals and blood-tonifying medicinals, and three qi-tonifying medicinals were identified (Figure 3).

## 4. Discussion

Traditional Korean Medicine has historical evidence accumulated over thousands of years. In the recent years, efforts are under way to modernize and reconstruct individual experiential knowledge and to improve the quality of Traditional Korean Medicine. The modernization of Traditional Korean Medicine means converting it into a reasonable and empirical system that fits the evidence-based medicine model, combining it with modern technology, modern academic thinking, and modern scientific culture [1, 2]. This may include experimental studies, observational studies, and clinical studies to determine the efficacy, effect, and mechanism of herbal medicine [3].

Infertility in a couple is defined as the failure to conceive after an arbitrary period of 12 months without the use of contraception. The infertility rate in Korea is estimated to be more than 13%, increasing every year [5], and there are an increasing number of patients seeking clinical evaluation and treatment for infertility [10].

Multiple treatment approaches are available for infertility, and assisted reproductive technology has become an important choice for infertile couple [10]. COH is a key determinant of the success of IVF-ET. However, the use of exogenous hormones at doses higher than the physiological dose reduces hormone levels leading to decreases in endometrial receptivity and perhaps also pregnancy outcomes [6, 15, 20–23].

In Korean Medicine clinics, herbal medicines have been widely used to prevent miscarriage and increase the implantation rate. Korean Medicine as an adjunctive therapy for assisted reproductive technology is effective in terms of induction of ovulation and development and maintenance of endometrium. In traditional Korean Medicine, pattern identification of infertility is divided into kidney deficiency, liver depression, dampness-phlegm, blood deficiency, and blood stasis. Clinically, the most common cases of infertility were in the category of kidney deficiency [24]. Recently, according to the menstrual cycle, treatment to help the recovery of the ovulation cycle is performed in the follicular phase, and supplement with function of the luteum to increase the rate of implantation is performed in the luteal phase [25]. The treatment for enhancing the function of the luteum is prescribed based on the tonifying kidney, such as Sutaehwan [24, 26]. However, there is no review of the experimental studies on how certain herbal medicine improves implantation.

Therefore as part of the study on modernization of Traditional Korean Medicine, we analyzed studies that clarified the effects of herbal medicines used to improve endometrial receptivity and promote pregnancy. Studies on implantation are conducted on animals for ethical reasons, so we investigated only experimental studies.

Through analyzing the publication year of 11 selected papers, the number of experimental studies on improvement of endometrial receptivity in herbal medicine tended to increase slightly, but most studies were published in China. In Korea, for the purpose of promoting the implantation, herbal medicine is widely used in clinical practice, and it should be backed up by experimental studies.

The herbal medicine and herbal medicinals which have tonifying effect were most commonly used in experimental studies, which is similar to herbal medicine used in the Korean Medicine clinic. These herbal medicines may improve endometrial receptivity by reinforcing the expression of endometrial LIF and integrin *β*. Among outcome measures, integrin *β*3 is a specific molecule for evaluating uterine receptivity during implantation, primarily expressed in the cytoplasm of endometrial glandular epithelial cells during the implantation window [15, 16]. Integrin is an important cell adhesion molecule that can recognize extracellular matrix proteins based on the integrin arginine-glycine-aspartic acid sequence, which mediates cell adhesion to the extracellular matrix, promotes production of angiogenesis factors, mediates intracellular and extracellular transduction, and increases the blood supply to the endometrium, thereby improving endometrial receptivity [27, 28]. LIF has been identified as a key mediator in promoting endometrial transformation. It can induce COX, enhance vascular permeability and angiogenesis, and reconstruct endometrial blood vessel [10]. Proper angiogenesis is fundamental to implantation, and pregnancy will not occur when the endometrial thickness is below a particular thickness [29]. Pinopodes are classical biomarkers of endometrial receptivity [30]. Nikas and Makrigiannakis reported that the coverage of pinopodes on the endometrial epithelium during the implantation window of normal fertile women is more than 50%, while women with a lower coverage of pinopodes, particularly those with a coverage <10%, tend to experience multiple implantation failures [31]. Proteomics is very important for understanding the action mechanism of traditional Chinese medicine. Li et al. suggested that these regulated proteins were important in regulating the uterine environment for the blastocyst implantation [12].

After reviewing these studies, we confirmed that herbal medicines have positive effects on factors associated with endometrial receptivity, leading to increase in endometrial receptivity and perhaps pregnancy outcomes. Therefore it is recommended that women who plan to become pregnant should be given treatment to improve the endometrial receptivity and to improve the implantation environment with the recovery of the ovulation cycle. In particular, when the endometrium is thin or repeatedly failed to be implantd, it is thought that Korean Medicine will be more necessary.

By confirming the enhancement of endometrial receptivity of herbal medicine in the COH model, we confirmed the use of herbal medicine to increase implantation rates by the adjunctive use of assisted reproductive technology. In particular, in two studies, herbal medicines have been shown to have a comparable effect compared to progynova and aspirin, which are used to promote implantation. As is known, the window of implantation moves forward during the controlled ovarian hyperstimulation, which negatively affects the implantation. If the Korean Medicine treatment which improves endometrial receptivity is performed together with COH, it may contribute to the increase of implantation rate.

However, these studies are limited to experimental research, and most were published in China. It is necessary to investigate the current use of prescriptions to enhance implantation in Korean Medicine clinics and to conduct experimental studies on the effects of those herbal medicines on endometrial receptivity. Reviews and experimental studies are needed as well to inform safety and efficacy in combination with Western Medicine prescribed in assisted reproductive technology. Furthermore, observational studies on the combined treatment effects of traditional Korean Medicine and assisted reproductive technology in the treatment of infertility are needed.

## 5. Conclusions

From 2008 to 2016, experimental studies on herbal medicine on endometrial receptivity yielded the following conclusions.

Experimental studies of the effects of herbal medicine on endometrial receptivity are gradually increasing. The experimental studies on herbal medicine on endometrial receptivity evaluate outcomes by two types of experimental models. The effects of herbal medicine used in experiments were invigorating kidney, replenishing Qi, nourishing blood, or activating blood. The most frequently used herbal medicinal materials were Astragali Radix and tonifying and replenishing medicinals. Herbal medicines appeared to improve endometrial receptivity in the EID or COH model and showed efficacy similar to progynova and aspirin.

Given that there is only domestic Korean study, evaluating the effectiveness of single herbal medicinal materials and studies on the prescription of clinical practice should begin. The safety and efficacy of combination of traditional Korean Medicine and Western Medicine for infertility should be studied. Observational studies on combination therapy for infertility treatment are also needed.

## Figures and Tables

**Table 1:** Summary of experimental studies on the improvement of endometrial receptivity in herbal medicine.

Number	Author	Study design	Control group	Experimental group	Outcome	Results	
(1)	Choi et al. [6]	In vivo: KM mice	Normal (*n* = 20) EID^*∗*^ (*n* = 23)	EID + TCM (*n* = 19)	Pregnancy rate	EID + TCM versus EID: ↑ (*P* < 0.05)	
					Implantation sites	EID + TCM versus EID: ↑ (*P* < 0.01)	
					LIF mRNA	EID + TCM versus EID on Pd 4, 6: ↑ (*P* < 0.01) EID + TCM versus EID on Pd 5 ↓ (*P* < 0.05) EID + TCM versus normal: not significant (*P* > 0.05)	
					LIF protein	EID + TCM versus Normal on Pd 6: not significant (*P* > 0.05)EID versus EID + TCM: ↓ (*P* < 0.05) EID versus normal: ↓ (*P* < 0.05)	

(2)	Xu et al. [10]	In vivo: KM mice	Normal (*n* = 12) EID^†^ (*n* = 12)EID + Progynova (*n* = 12)	EID + TCM (0.91 g/Kg) (*n* = 12) EID + TCM (1.82 g/Kg) (*n* = 12) EID + TCM (3.64 g/Kg) (*n* = 12)	PPARd, IL-11 mRNA	TCM at doses of 1.82 g/kg, 3.64 g/kg versus progynova: ↑ (*P* < 0.05)	
					COX-2	In gland	EID + TCM, EID + Progynova versus EID: ↑ (*P* < 0.05) EID + TCM versus Progynova: ↑ (*P* > 0.05)
						In stroma	not significant (*P* > 0.05)
					PGI2	In gland	EID + TCM, EID + Progynova versus EID: ↑ (*P* < 0.05) EID + TCM at high doses versus EID + Progynova: ↑ (*P* < 0.05)
						In stroma	EID + TCM versus EID: ↑ (*P* < 0.05)
					MMP-9	In gland	EID + TCM, EID + Progynova versus EID: ↑ (*P* < 0.05)
						In stroma	EID + TCM, EID + Progynova versus EID: ↑ (*P* < 0.05)
					TIMP-3	In gland	EID + TCM, EID + Progynova versus EID: ↑ (*P* < 0.05) EID + TCM at high dose versus EID + Progynova: ↑ (*P* < 0.05)
						In stroma	not significant (*P* > 0.05)

(3)	Cui et al. [14]	In vivo: KM mice	Normal (*n* = 10) COH^‡^ (*n* = 10)	COH + TCM (BS) (*n* = 10) COH + TCM (HX) (*n* = 10) COH + TCM (BH) (*n* = 10)	Endometrial thickness	COH + BS^§^, COH + HX^||^, COH + BH^¶^ versus COH: ↑ (*P* = 0.0004, <0.0001, <0.0001) COH + BH^¶^ vs COH + BS^§^: ↑ (*P* = 0.02)	
					Microvessel density	COH + BS^§^, COH + HX^||^, COH + BH^¶^ versus COH: ↑ (*P* = 0.0046, 0.0003, 0.0004)	
					Pinopodes in the epithelium	COH + HX^||^, COH + BH versus COH: ↑ (*P* = 0.0011, 0.0009) COH + HX^||^, COH + BH versus COH + BS^§^: ↑ (*P* = 0.0374, 0.033)	

(4)	Huang et al. [11]	In vivo: KM mice	Normal (*n* = 30) EID^*∗∗*^ (*n* = 30)	EID + TCM (*n* = 30)	Pregnancy rate	EID + TCM versus EID: ↑ (*P* < 0.05)	
					Number of implanted blastocysts	EID + TCM versus EID: ↑ (*P* < 0.01)	
					Proliferative index	In gland	EID + TCM versus EID: ↓ (*P* < 0.01) EID + TCM versus normal: not significant (*P* > 0.05)
					Proliferative index	In stroma	not significant (*P* > 0.05)
					Apoptotic index	In gland	EID + TCM versus EID: ↑ (*P* < 0.01) EID + TCM versus normal: not significant (*P* > 0.05)
					Apoptotic index	In stroma	not significant (*P* > 0.05)

(5)	Sun et al. [15]	In vivo: KM mice	Normal (*n* = 5) COH^‡^ (*n* = 5)	COH + TCM (*n* = 5)	Integrin *β*3 mRNA	COH + TCM versus COH on Pd 2, 4: ↓ (*P* < 0.01) Among 3 groups on Pd 0, 6, 8: not significant (*P* > 0.05)	
					OPN mRNA	COH + TCM versus COH on Pd 4, 6, 8: ↓ (*P* < 0.01) Among 3 groups on Pd 0, 2: not significant (*P* > 0.05)	

(6)	Chen et al. [16]	In vivo: KM mice	Normal (*n* = 10) COH^‡^ + aspirin (*n* = 30)	COH + TCM (*n* = 30)	Integrin *β*3 mRNA	COH + TCM versus aspirin: ↑ (*P* < 0.01)	
					LIF mRNA/*β*-actin	COH + TCM versus aspirin: ↑ (*P* = 0.02)	

(7)	Yu et al. [18]	In vivo: KM mice	Normal (*n* = 18) COH^‡^ (*n* = 15)EID^*∗∗*^ (*n* = 16)	TCM (*n* = 21)COH + TCM (*n* = 22) EID + TCM (*n* = 23)	Pregnancy rate	COH + TCM versus COH: ↑ (*P* < 0.01) EID + TCM versus EID: ↑ (*P* < 0.01) TCM versus normal: not significant difference (*P* > 0.05)	
					Implantation sites	TCM versus EID: ↑ (*P* < 0.05) EID + TCM versus EID: ↑ (*P* > 0.05)	
					LIF proteins	TCM versus COH, EID: ↑ (*P* < 0.05, *P* < 0.01)	
					Integrin *β*3 proteins	COH + TCM, EID + TCM versus COH, EID: ↑ (*P* < 0.01, *P* < 0.05)	
					LIF mRNA	TCM versus COH + TCM: ↑ (*P* < 0.05) TCM versus EID + TCM: ↑ (*P* < 0.05)	
					Integrin *β*3 mRNA	TCM versus COH + TCM: ↑ (*P* < 0.05) TCM versus EID + TCM: ↑ (*P* < 0.05)	

(8)	Li et al. [12]	In vivo: KM mice	Normal (*n* = 6) COH^*∗*^ (*n* = 6)	COH + TCM (*n* = 6)	LCM-DE-MS^††^	COH + TCM group showed significant changes in 23 proteins: 7 proteins downregulated^‡‡^ and 16 proteins upregulated^§§^	

(9)	Wu et al. [13]	In vivo: KM mice	Normal (*n* = 10) EID^*∗*^ (*n* = 10)	EID + TCM (*n* = 10)	Pregnancy rate	EID + TCM versus EID: ↑ (*P* < 0.05) EID + TCM versus normal: ↓ (*P* < 0.05)	
					Number of Implanted embryos	EID + TCM versus EID: not significant (*P* > 0.05) EID + TCM versus normal: ↓ (*P* < 0.05)	
					NF-*κ*В	EID versus Normal on Pd 5, 6, 7: ↑ (*P* < 0.01) EID versus EID + TCM on Pd 5, 6, 7: ↑ (*P* < 0.01) EID + TCM versus normal during whole time: not significant (*P* > 0.05)	
					IFN-*γ*, IL-10	EID + TCM versus normal: not significant (*P* > 0.05) EID + TCM versus EID on Pd 5, 6, 7: ↑ (*P* < 0.01)	

(10)	Choi et al. [9]	In vitro: Ishikawa cells			LIF mRNA	PL-PP group showed significant increase dose-dependently (*P* < 0.05)	
					Adhesion of JAr spheroids	PL-PP versus Control: ↑ (*P* < 0.05)	
		In vivo: KM mice	Normal (*n* = 11) EID^*∗∗*^ (*n* = 11)	EID + PL-PP (*n* = 11)	No of implanted embryos	PL-PP versus RU486: ↑ (*P* < 0.05) PL-PP versus normal: ↑ (*P* < 0.05)	

(11)	Li et al. [17]	In vivo: KM mice	Normal (*n* = 60) COH^‡^ (*n* = 60)	TCM (*n* = 60)	Number of implanted blastocyst	TCM + model versus model: ↑ (*P* < 0.05)	
					Integrin *αν* mRNA	Model versus TCM, control: ↓ (*P* < 0.05)	
					Integrin *β*3 mRNA	Model versus TCM, control: ↓ (*P* < 0.05)	

^*∗*^Indomethacin; ^†^Hydroxyurea + Mifepristone; ^‡^Pregnant mare serum gonadotropin (PMSG) + human chorionic gonadotropin (hCG); ^§^BS: *Bushen (Semen Cuscutae, Herba Taxilli Chinensis, Radix Dipsaci)*; ^||^HX: *Huoxue (Radix Astragali, Radix Angelicae Sinensis, Radix Salviae)*; ^¶^BH: *Bu-Shen-An-Tai*; ^*∗∗*^Mifepristone; ^††^Laser capture microdissection-double dimensional electrophoresis-mass spectrum; ^‡‡^collagen *α*-1 (VI) chain, keratin 7, keratin 14, myosin regulatory light chain 12B, myosin light polypeptide 9, heat shock protein *β*-7, and C-U-editing enzyme APOBEC-2; ^§§^apolipoprotein A-I, calcium regulated protein-3, proliferating cell nuclear antigen, L-xylulose reductase, and calcium binding protein.

**Table 2:** Composition of prescription.

No	Prescription	Medicinal materials
(1)	*Bushenantai* [6]	*Cuscutae Semen, Loranthi Ramulus et Folium, Dipsaci Radix, Salviae Miltiorrhizae Radix, Astragali Radix, Angelicae Gigantis Radix*

(2)	PL-PP [9]	Polysaccharides depleted-water extract of *Paeonia lactiflora Pallas *

(3)	*Xianziyizhen* [10]	*Rehmanniae Radix, Ligustici Fructus, Ecliptae Herba, Dipsaci Radix, Cuscutae Semen, Epimedii Herba*

(4)	*Bushenyiqihexue* [11]	*Loranthi Ramulus et Folium, Salviae Miltiorrhizae Radix, Astragali Radix, Angelicae Gigantis Radix, Cnidium officinale Makino*

(5)	DS-1-47 [12]	*Astragali Radix, Atractylodes macrocephala Koidzumi, Scutellariae Radix, *Dipsacales

(6)	*Bangdeyun* [13]	*Dipsaci Radix, Astragali Radix, Salviae Miltiorrhizae Radix, Scutellariae Radix, Atractylodes macrocephala Koidzumi*

(7)	*BuShenAnTai* [14]	*Cuscutae Semen, Loranthi Ramulus et Folium, Dipsaci Radix, Astragali Radix, Angelicae Gigantis Radix, Salviae Miltiorrhizae Radix*

(8)	*Er'zhi Tiangui* [15]	*Lycium chinense Miller, Ligustici Fructus, Ecliptae Herba, Cuscutae Semen, Angelicae Gigantis Radix, Paeonia lactiflora Pallas, Cnidium officinale Makino, Rehmanniae Radix, Cyperi Rhizoma, Glycyrrhiza uralensis Fischer and so forth*

(9)	*Shoutaiwai* [16]	*Astragali Radix, Loranthi Ramulus et Folium, Dipsaci Radix, Cnidium officinale Makino, Atractylodes macrocephala Koidzumi, Adenophorae Radix*

(10)	*Yiqixue Buganshen* [17]	*Astragali Radix, Adenophorae Radix, Atractylodes macrocephala Koidzumi, Eucommiae Cortex, Glycyrrhiza uralensis Fischer, Rehmanniae Radix, Cinnamomi Cortex, Angelicae Gigantis Radix, Paeonia lactiflora Pallas, Cnidium officinale Makino*

(11)	*Zhuyun* [18]	*Epimedii Herba, Morindae Radix, Cuscutae Semen, Eucommiae Cortex*
